# Ultrasound-Guided Real-Time Visual Biofeedback for Internal Impingement in a Collegiate Baseball Player: A Case Report

**DOI:** 10.7759/cureus.94416

**Published:** 2025-10-12

**Authors:** Koji Wagatsuma, Masashi Kawabata, Hiroyuki Kobayashi, Tomoki Miwa, Wataru Iwamoto

**Affiliations:** 1 Department of Rehabilitation, Jinseikai, Tokyo, JPN; 2 Department of Rehabilitation, Kitasato University School of Allied Health Sciences, Sagamihara, JPN; 3 Department of Rehabilitation, Medical Base Shinkoiwa, Tokyo, JPN; 4 Department of Sports Medicine, Edogawa Hospital, Tokyo, JPN

**Keywords:** baseball, internal impingement, overhead athlete, rotator cuff, shoulder impingement, ultrasound-guided, visual biofeedback

## Abstract

Shoulder impingement syndrome is highly prevalent among overhead athletes, with rehabilitation typically requiring three to six months before returning to the sport. A 20-year-old male collegiate baseball outfielder (right-hand pitcher, left-hand batter) developed right-shoulder pain during throwing and was diagnosed with internal impingement two months after onset. Pain occurred in the early cocking phase, with 0° internal rotation at 90° flexion and abduction limited to 140°. Pain was induced by 90° abduction [numeric rating scale (NRS): 8/10]. The strength of the infraspinatus and teres minor was reduced, and the Neer, Hawkins, and Hornblower tests were positive. Dynamic ultrasonography revealed impaired teres minor contractions with compensatory posterior deltoid activation. Treatment included manual range-of-motion and flexibility exercises, ultrasound-guided real-time visual biofeedback once weekly to facilitate selective teres minor activation, and a structured at-home program emphasizing daily repetitions of teres minor contractions. Active external rotation in the elevated arm position improved after two weeks, and teres minor strength increased, allowing the initiation of a return-to-throw program. At four weeks, the NRS score decreased from 8/10 to 3/10, and the patient was able to throw 20 m. He achieved his pre-injury performance level at six weeks following five therapy sessions, and returned to competition before the league season. At the three-month follow-up, the patient remained asymptomatic and continued to play without any recurrence. Ultrasound-guided real-time visual biofeedback accelerated the player’s return to the sport despite shoulder impingement symptoms. Our approach can serve as a valuable adjunct to conventional rehabilitation, potentially reducing loss of time in overhead athletes.

## Introduction

Shoulder impingement syndrome is highly prevalent among overhead athletes, with internal impingement being the primary source of pain in baseball players. Internal impingement occurs when the articular-side rotator cuff repetitively contacts the posterosuperior labrum in abducted and externally rotated positions [1]. Overuse-related shoulder injuries are a common cause of loss of time in collegiate baseball and rank among the top two injuries estimated across programs [2]. Rotator cuff-targeted, exercise-based rehabilitation has been shown to reduce pain [3]. Among individuals with chronic symptoms persisting for three months or longer, three distinct exercise approaches have achieved pain reduction [4]. However, the overall quality of the evidence remains low, relying largely on expert opinion rather than high-level trials [5], and conservative rehabilitation typically requires three to six months [1,6]. Cha et al. reported improvements in pain and strength balance after a 12-week program for pitchers with shoulder impingement [7]. Although return-to-play can be achieved with conservative treatment, the prolonged time course remains a major limitation for competitive athletes. To the best of our knowledge, no previous studies have described the application of ultrasound-guided real-time visual biofeedback as a strategy to facilitate early return-to-play in baseball players with shoulder impingement symptoms. The present case illustrates how this approach enabled a successful return to competition within six weeks.

## Case presentation

A 20-year-old male collegiate baseball outfielder (right-hand pitcher and left-hand batter) developed right shoulder pain while throwing during a game. Symptoms began approximately two months before presentation. Despite persistent pain, the patient had continued to play and had not received any treatment. He wished to return before the collegiate league season that was scheduled three months later.

**Figure 1:**
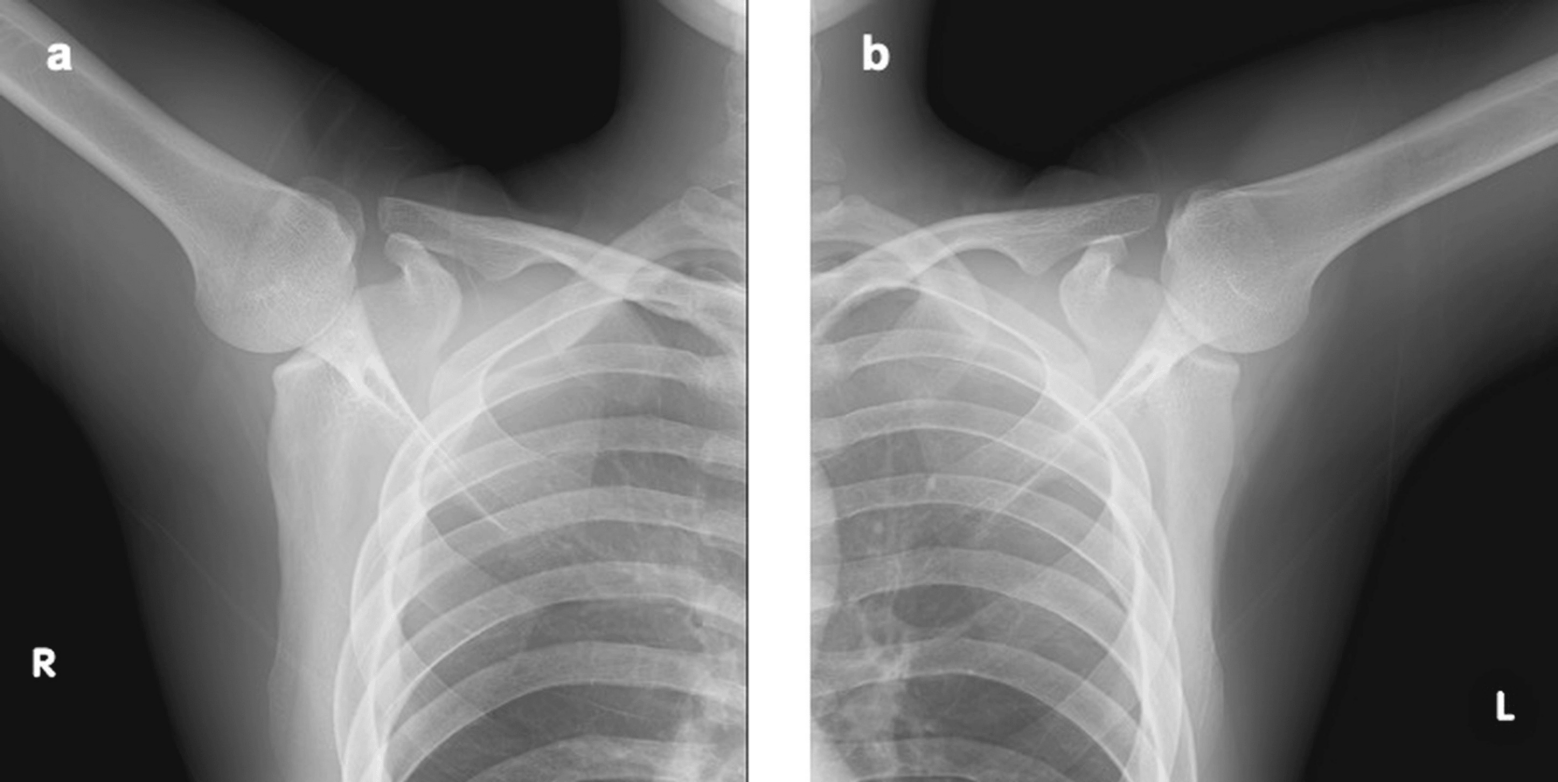
Plain radiographs of the shoulder. Upright radiographs obtained with the arm in an elevated-arm position showed no abnormalities. a) right shoulder, b) left shoulder.

Plain radiographs obtained in an elevated arm position were unremarkable (Figure 1). Magnetic resonance imaging (MRI) was not performed because the clinical and dynamic US findings were considered sufficient for diagnosis. Differential diagnoses such as glenohumeral instability, superior-labrum-anterior-to-posterior tear lesions, and labral tears were excluded based on negative apprehension and relocation tests, absence of mechanical symptoms, and unremarkable radiographs. The patient was diagnosed with right internal impingement.

Physical therapy was initiated to reduce the throwing-related pain. The baseline physical examination revealed 0° internal rotation at 90° flexion and abduction limited to 140°. Pain appeared at 90° of abduction with an NRS score of 8/10. Pain intensity was assessed using the numeric rating scale (NRS), which ranges from 0-10, is freely available, and does not require a license [8].

**Figure 2:**
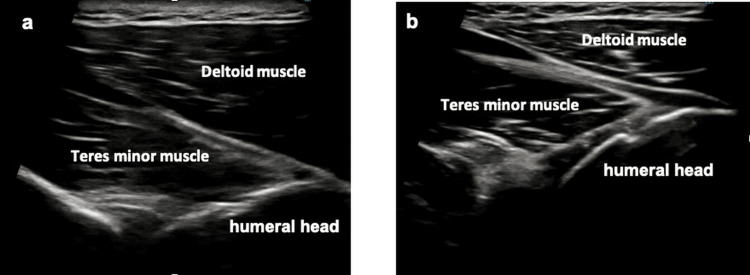
Dynamic ultrasound (US) assessment of the teres minor. a) affected side, b) unaffected side.

The strength of the infraspinatus and teres minor muscles was reduced (manual muscle testing: 4/5). Muscle strength was evaluated using the manual muscle testing (MMT) scale, which ranges from 0-5, is freely available, and does not require a license [9]. The results of Neer, Hawkins, and Hornblower tests were positive (performed according to their original descriptions [10-12]. Analysis of the throwing motion reproduced pain in the back of the shoulder during the early cocking phase, accompanied by compensatory early scapular elevation. Dynamic US demonstrated a larger change in thickness in the posterior deltoid than in the teres minor, indicating impaired contraction of the teres minor (Figure 2, Video 1). Based on these findings, teres minor dysfunction in the elevated arm position was considered to contribute to the impingement.

**Video 1:** Dynamic ultrasound (US) assessment of the teres minor. Dynamic US demonstrating a greater thickness change in the posterior fibers of the deltoid than in the teres minor on the affected side, indicating weak teres minor contraction. a) affected side: larger thickness change of the posterior deltoid compensating for external rotation normally produced by the teres minor. b) unaffected side: minimal deltoid contraction with selective teres minor contraction.

Treatment consisted of a manual range-of-motion and flexibility exercises, US-guided real-time visual biofeedback (20 min, once weekly) to facilitate selective teres minor activation (Video 2), and a structured home exercise program emphasizing teres minor contraction (three sets of 10 repetitions daily). Posterior capsule stretching, scapular stabilization exercises for the serratus anterior and lower trapezius, and manual therapy to improve glenohumeral mobility were performed. The patient demonstrated high adherence to the prescribed home program and reported daily performance without missed sessions, which likely contributed to a favorable recovery timeline.

**Video 2:** Dynamic ultrasound (US)-guided real-time visual biofeedback therapy. In addition to manual range-of-motion exercises, US-guided real-time visual biofeedback was used to facilitate selective teres minor activation. The scapula was first protracted and stabilized via serratus anterior action. With the shoulder flexed to 90°, the arm was contracted into end-range external rotation. Repetitions were performed while visually confirming teres minor contraction on the US image.

**Figure 3:**
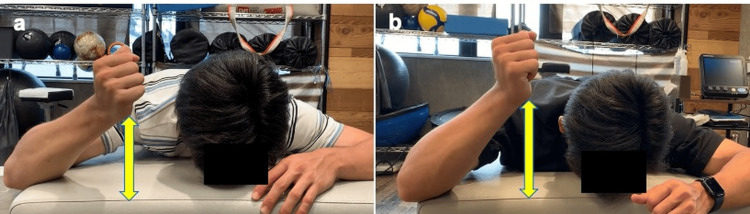
Teres minor: pre- and post-treatment comparison. Active external rotation in the elevated-arm position increased, and teres minor strength improved. a) active external rotation angle in the elevated position at baseline. b) active external rotation angle in the elevated position at one week

One week after treatment initiation, pain during shoulder abduction improved, active external rotation in the elevated arm position increased, and teres minor strength showed clear improvement (Figure 3). A return-to-throw program was initiated, although mild pain persisted during the early cocking phase. At week four, the NRS pain score decreased from 8/10 to 3/10, and the patient was able to throw approximately 20 m without any major discomfort. By week five, the pain during throwing had resolved, allowing the resumption of full team practice. After five treatment sessions, he achieved his target performance level at week six and successfully returned to competitive play before the start of the league season. At the three-month follow-up, the athlete remained asymptomatic and continued to compete without pain recurrence or functional limitations.

## Discussion

This report demonstrates that US-guided real-time visual biofeedback facilitates early return to sports in collegiate baseball players with shoulder impingement symptoms. Although no previous reports have described this approach for baseball-related internal impingement, previous studies have investigated US-guided feedback for other muscle groups [13]. In particular, adding real-time US visual biofeedback significantly increases serratus anterior activity compared to manual therapy alone. A systematic review reported that rehabilitative US imaging used as visual biofeedback during motor control exercises was more effective than verbal or tactile feedback for enhancing muscle activation and task performance [14]. These findings support the clinical rationale for using US-guided feedback in overhead athletes.

The therapeutic effect in this patient likely involved the restoration of teres minor function. Cadaveric studies indicate that the teres minor muscle, along with the other rotator cuff muscles, counteract the superior translation of the humeral head and impart an inferior shear force, acting as a principal humeral head depressor during elevation [15-17]. Improved teres minor activation reduces impingement and facilitates early pain relief. Experimental studies have demonstrated that pain inhibits muscle output, whereas pain alleviation rapidly restores strength [18,19]. In this patient, US-guided visual biofeedback may have enhanced selective teres minor activation and suppressed pain-related inhibition, leading to early strength gain and an accelerated return to competition.

The novelty of this report lies in the shortened rehabilitation period. Previous studies suggested that a recovery period of 12 weeks or longer is typically required for pitchers with shoulder impingement [7]. However, this patient returned to competitive play within six weeks. This suggests that incorporating US-guided visual biofeedback into conventional rehabilitation may represent a practical strategy for reducing loss of time in competitive athletes.

This study had some limitations, in that it was a single-patient observation that relied primarily on clinical findings and patient-reported assessments without objective measures, such as dynamometer-based strength testing. Additionally, an MRI was not performed, although major differential diagnoses were excluded clinically and radiographically. Larger systematic studies are required to verify the effectiveness, reproducibility, and long-term outcomes of this intervention.

## Conclusions

Rotator cuff contraction training delivered with ultrasound-guided real-time visual biofeedback enabled early return to sports despite shoulder impingement symptoms. Overall, this experience suggests that ultrasound-guided visual biofeedback may be a valuable adjunct to conventional rehabilitation in overhead athletes, potentially accelerating recovery and reducing time loss from competition. Future studies involving larger samples are warranted to validate this approach.
